# Cellular and Humoral Immune Responses to Vaccination for COVID-19 Are Negatively Impacted by Senescent T Cells: A Case Report

**DOI:** 10.3390/vaccines11040840

**Published:** 2023-04-14

**Authors:** Eliane Aparecida Rosseto-Welter, Silvia Sanches Rodrigues, Amanda Braga de Figueiredo, Carolina Nunes França, Danielle Bruna Leal Oliveira, André Luis Lacerda Bachi, Jônatas Bussador do Amaral, Ricardo Andreotti Siqueira, Laiz Camerão Bento, Ana Paula da Silva, Nydia Strachman Bacal, Carlos Eduardo dos Santos Ferreira, Cristóvão Luis Pitangueira Mangueira, João Renato Rebello Pinho

**Affiliations:** 1 Hospital Israelita Albert Einstein, São Paulo 05652-900, Brazil; 2 Post-Graduation Program in Health Sciences, Santo Amaro University, São Paulo 04829-300, Brazil; 3 Departmento de Microbiologia, Instituto de Ciências Biomédicas, Universidade de São Paulo, São Paulo 05508-900, Brazil; 4 ENT Research Lab., Department of Otorhinolaryngology Head and Neck Surgery, Federal University of Sao Paulo (UNIFESP), São Paulo 04021-001, Brazil; 5 LIM 03/07, Faculdade de Medicina, Universidade de São Paulo, São Paulo 05403-010, Brazil

**Keywords:** CoronaVac vaccine, Pfizer vaccine, immunophenotyping, inflammation, neutralizing antibodies, cell senescence

## Abstract

Background: Herein, we aimed to follow up on the cellular and humoral immune responses of a group of individuals who initially received the CoronaVac vaccine, followed by a booster with the Pfizer vaccine. Methods: Blood samples were collected: before and 30 days after the first CoronaVac dose; 30, 90, and 180 days after the second CoronaVac dose, and also 20 days after the booster with the Pfizer vaccine. Results: Whilst the positivity to gamma interferon-type cellular response increased after the first CoronaVac dose, neutralizing and IgG antibody levels only raised 30 days after the second dose, followed by a drop in these responses after 90 and 180 days. The booster with the Pfizer vaccine elicited a robust cellular and humoral response. A higher number of double-negative and senescent T cells, as well as increased pro-inflammatory cytokines levels were found in the participants with lower humoral immune responses. Conclusion: CoronaVac elicited an early cellular response, followed by a humoral response, which dropped 90 days after the second dose. The booster with the Pfizer vaccine significantly enhanced these responses. Furthermore, a pro-inflammatory systemic status was found in volunteers who presented senescent T cells, which could putatively impair the immune response to vaccination.

## 1. Introduction

SARS-CoV-2, the new coronavirus responsible for the COVID-19 infection, was first detected in Wuhan, China, at the end of 2019, and, at the time of writing this manuscript, is responsible for a pandemic with more than 758 million confirmed cases and 6.85 million deaths reported by the WHO [1,2,3].

Among several aspects of the COVID-19 pandemic, one that deserves to be highlighted is associated with the rapid production and distribution of COVID-19 vaccines. In fact, a massive vaccination campaign has been performed around the world, since it was accepted that the SARS-CoV-2 vaccination is the best option to achieve global population protection against COVID-19 and, perhaps, to put an end to the pandemic [4,5,6].

However, it is important to mention that the mechanisms associated with the protective response elicited by different schedules of vaccination, especially with the involvement of the CoronaVac vaccine, the first vaccine applied in the Brazilian population, are not fully understood. CoronaVac (BBIBP-CorV, Sinovac Biotech) is composed of inactivated whole SARS-CoV-2 virus [7,8]. This vaccine was initially used in two doses, in which the second dose of vaccine was applied around 28 days after the first dose, and it is currently produced in the “Instituto Butantan”, belonging to the São Paulo Government, Brazil.

It has been reported that CoronaVac is able to elicit a robust antibody response to COVID-19, which could be associated with a reduction in hospital admissions and COVID-19-related death [9]. However, the antibody response induced by two doses of CoronaVac significantly decreases over time [10]. It was also demonstrated that two doses of the CoronaVac vaccine presented the capacity to induce a robust cellular response, predominantly by CD4+ T cells polarized towards a Th1 immune profile [11]. Due to the fact that whether this cellular response is maintained over time was poorly understood, a third dose of vaccine for COVID-19 was offered. From this moment, it was possible to use a different type of vaccine to those previously received, in order to boost the immune responses elicited. In this sense, in Brazil, the vaccines available to be used as the third dose for individuals previously vaccinated against COVID-19 exclusively with CoronaVac included the Pfizer vaccine (BNT162b2 mRNA from BioNTech).

Based on this information, in the current case report, we aim to increase the knowledge concerning both the humoral and cellular responses in a group of collaborators at Hospital Israelita Albert Einstein in São Paulo, Brazil, submitted to a schedule for COVID-19 vaccination, in which all volunteers were initially submitted to the CoronaVac vaccine, followed by a booster with the Pfizer vaccine.

## 2. Detailed Case Description

### 2.1. Materials and Methods

A group of eight volunteers were followed up for their immune response against SARS-CoV-2 in different conditions after vaccination: pre-vaccination; between the first two doses; approximately 30, 90, and 180 days after the second dose; and 30 days after the booster dose with the Pfizer vaccination. It should be mentioned that the number of volunteers enrolled in this study was related not only to the fact that these individuals were submitted to the same vaccination schedule, but also to the fact that their samples were submitted to all the methods presented in this study. The volunteers received all the information about the study and were oriented to sign the informed consent form, previously approved by the Ethics Committee of the Hospital Israelita Albert Einstein (approval number 4.159.565). The study is in agreement with the guidelines of the Declaration of Helsinki.

The ELISpot (Enzyme Linked ImmunoSpot) technique was used for the qualitative detection of specific COVID-19 interferon-gamma (IFN-γ) released by peripheral blood mononuclear cells (PBMC) using T-SPOT SARS-CoV-2 kits (Oxford Immunotec Ltd., Abingdon, UK) with S and N panels. Reactivity was determined by the final spot count: reactive if ≥8 points; borderline, from 5 to 7 points; not reactive, ≤4 points.

In the evaluation of the humoral response, the tests used to search for total neutralizing antibodies against-SARS-CoV-2 in serum were: (1) cPass™ SARS-CoV-2 Neutralization Antibody Detection (GenScript Biotech Corporation, Nanjing, China), with results ≥ 30% considered reactive; (2) Cytophatic Effect Virus Neutralization Test (CPE-VNT), performed as the gold standard in the Universidade de São Paulo, Instituto de Ciências Biomédicas (ICB-USP), with titers ≥ 1/20 considered reactive; (3) the IgG response to RBD (receptor binding domain) of the viral spike protein was quantified in serum using the automated chemiluminescent assay (CMIA) SARS-CoV-2 IgG II Quant (Abbott Ireland Diagnostics Division); results ≥ 7.1 BAU/mL were considered positive.

For the quantification and characterization of lymphocytes by flow cytometry, we used the DuraClone IM T cell subsets kit (Beckman Coulter, Brea, CA, USA) in whole blood with EDTA, which included the following markers: CD45RA FITC (2H4), CD197 (CCR7) PE(G043H7), CD28 ECD (CD28.2), CD279 (PD) PC5.5 (PD1.3.5), CD27 PC7 (1A4.CD27), CD4 APC (13B8.2), CD8 Alexa Fluor 700 (B9.11), CD3 Alexa Fluor A750 (UCHT-1), CD57 Pacific Blue (NC1), and CD45 Krome Orange (J33). At least 100.000 events were acquired in a NAVIOS flow cytometer. Through FlowJo™ software (v 10.8.1) analyses, total lymphocytes from all volunteers were concatenated and submitted to a novel manifold learning technique for dimension reduction (UMAP—uniform manifold approximation and projection). This algorithm is used to visualize cell clusters according to the similarity of expression of all evaluated markers, allowing for identification of differentially frequent populations between the study groups, including unknown populations that could be ignored in conventional gate strategies. Finally, we correlated humoral response data pre-vaccine between the two doses and approximately 90 days after the second dose of Coronavac.

The serum levels of the cytokines TNF-α, IL-6, and IL-10 were determined by ELISA kits (Thermo Fisher Scientific, Invitrogen, Vienna, Austria), following the manufacturer’s instructions. In addition, the ratio between pro-inflammatory (TNF-α and IL-6) and anti-inflammatory (IL-10) cytokines was also calculated.

It is important to mention that saliva samples were collected at the time point of blood sampling and used to assess the SARS-CoV-2 diagnosis through the real-time PCR (RT-PCR) test. Based on this test, none of the volunteers presented SARS-CoV-2 infection.

In relation to the statistical analysis, it is important to point out that, as we separated the volunteer group into two subgroups according to their responsiveness to COVID-19, it was impossible to perform a statistical analysis, since the non-responder subgroup was composed of only two volunteers.

### 2.2. Results

Table 1 shows the qualitative detection of specific COVID-19 interferon-gamma (IFN-γ), with S and N panels, assessed using the ELISpot technique. It can be observed that the first dose of the CoronaVac vaccine elicited a rapid and specific IFN-γ response for COVID-19 in at least half of the participants in this study. In addition, although the specific IFN-γ response for COVID-19 was variable at other time-points associated with CoronaVac, after booster vaccination (with Pfizer vaccine), all volunteers showed a significant increase in this response.

**Figure 1:**
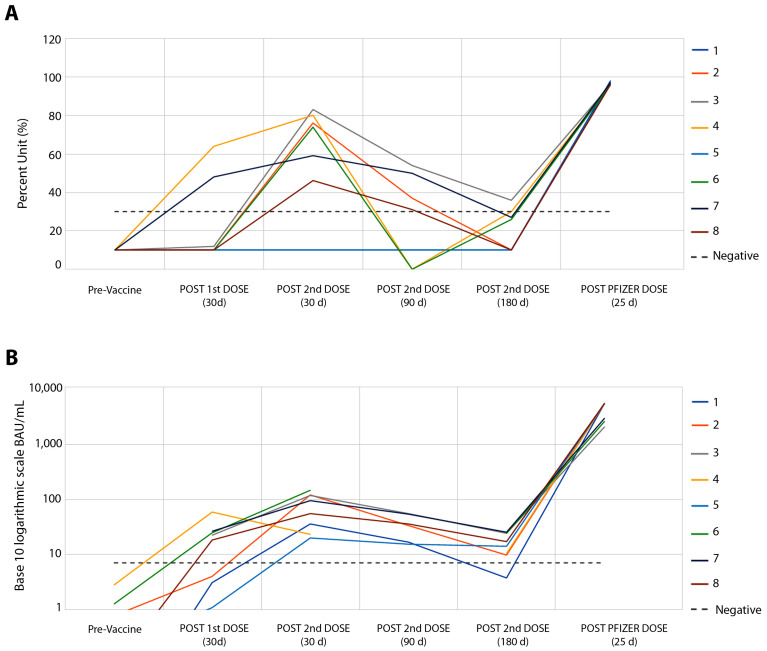
Follow-up of eight volunteers from pre-CoronaVac vaccine to Pfizer booster. (**A**) SARS-CoV-2 Surrogate Virus % Neutralization Test, sVNT (cPass™ GenScript USA, Inc., Piscataway NJ, USA). There was no difference between the results of neutralizing antibody tests between the sVNT and CPE-VNT methods, *p* > 0.131. (**B**) Automated chemiluminescent assay (CMIA) SARS-CoV-2 IgG II Quant, performed on the ARCHITECT i System (Abbott Ireland Diagnostics Division).

Figure 1 shows the results concerning the serum levels of total neutralizing antibodies against SARS-CoV-2 (Figure 1A) and the IgG response to RBD (Figure 1B). Higher levels of neutralizing antibodies and IgG were observed 30 days after the second dose of the CoronaVac vaccine, in which the relationship between antibody levels and their reference values showed a median of 2.25 times for neutralizing antibodies (Figure 1A) and a median of 10.7 times for IgG antibodies (Figure 1B). Similar to that observed in the IFN-g response, 90 and 180 days after the second dose of this vaccine, humoral responses showed a drop in levels, with negative rates of neutralizing antibodies in six of the eight volunteers (Figure 1A), and IgG response in all volunteers (Figure 1B). A total of twenty-three days after the booster with the Pfizer vaccine, a robust humoral response was found, ranging from 2.6 to 9.8 times more for neutralizing antibodies (Figure 1A) and from 87 to 1509 times more for IgG response (Figure 1B).

**Figure 2:**
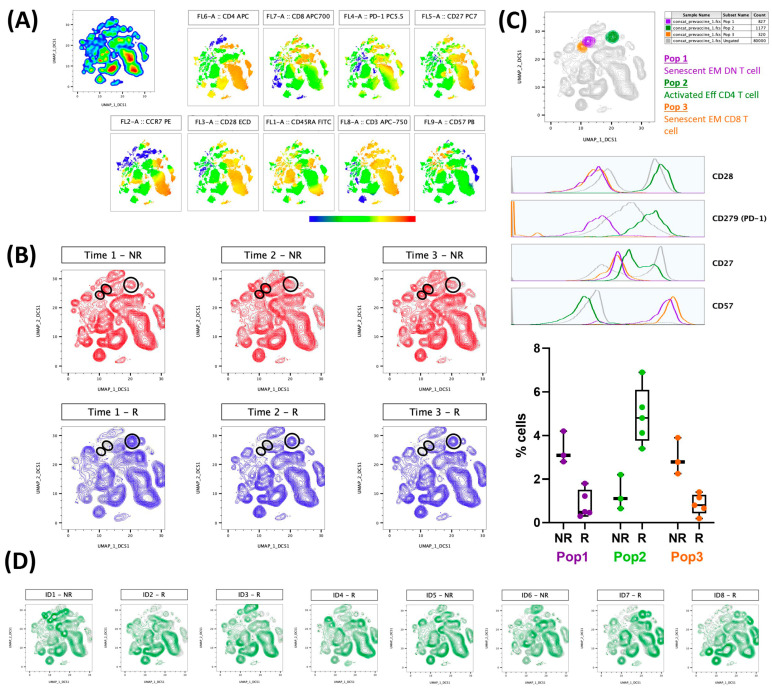
Activated T cells are related to the production of neutralizing antibodies, and, on the other hand, senescent T cell populations can impair this production by volunteers vaccinated with CoronaVac. Whole blood collected before first Coronavac dose was analyzed by flow cytometry. Analysis were performed using FlowJo software. A total of 10,000 lymphocytes from each volunteer were concatenated before performing unsupervised analysis. Neutralizing antibody production was evaluated after first (22 to 34 days, time 1), second (30 to 38 days, time 2), and 90 days (88 to 103 days, time 3) after second dose Coronavac vaccine. (**A**) Colormap graphs for lymphocytes populations present in whole blood from volunteers before Coronavac vaccination. (**B**) UMAP graphs showing volunteers that produced (R—blue) or did not produce (NR—red) neutralizing antibodies after Coronavac vaccination. (**C**) Top: UMAP graph and histograms showing the cell populations found in responder (Pop 2—green) or non-responder (Pop 1—purple and Pop 3—orange) volunteers. Down: Frequencies of populations 1-3 for R and NR 90 days after second dose Coronavac vaccine, including individual values, median, and interquartile range. (**D**) UMAP graphs representative of each volunteer individually, considering R and NR 90 days after second dose Coronavac vaccine. EM = effector memory; Eff = effector; DN = double-negative.

Figure 2 presents the results regarding the evaluation of lymphocyte population immunophenotyping (activated (CD27+CD28+CD57−CD297+) or senescent (CD27−CD28−CD57+)) in association with the results obtained in the neutralizing antibody responses. Figure 2A shows the colormap graphs (highlighting the surface markers used in the immunophenotyping) for the lymphocyte population present in whole blood from volunteers before the CoronaVac vaccination, whereas Figure 2B shows the volunteers that produced (responders—blue) or did not produce (non-responders—red) neutralizing antibodies after the CoronaVac vaccination. Figure 2C shows not only the compilation of the distribution of activated (orange color) and senescent (purple and orange) lymphocyte populations (superior panel), but also the representative histograms of each surface marker used in this study, as well as the frequencies of cells (activated and senescent) in the volunteer groups, both responders (R) and non-responders (NR), 90 days after the second dose of the CoronaVac vaccine. The purple gate is related to the senescent effector memory double negative T cell (CD4−CD8−) population, the orange is the senescent effector memory CD8+ T cell population, and the green is the activated effector CD4+ T cells. Of note, these populations were found using an unsupervised analysis, with UMAP, a machine learning technique, and by not searching previously characterized populations. Figure 2D presents the results obtained from each volunteer, highlighting that the graphs correspond to the non-responder volunteers, and the other graphs correspond to the responder volunteers.

Based on the fact that the two non-responder volunteers presented a higher frequency of senescent T cells than the responder volunteers, we performed an evaluation of the systemic inflammatory status of all participants. As presented in Table 2, interestingly, the two non-responder volunteers showed not only increased serum levels of the pro-inflammatory cytokines IL-6 and TNF-α, but also a higher ratio between IL-6 and IL-10 (IL-6/IL-10) and TNF-α and IL-10 (TNF-α/IL-10) than the values found in the responder group. Regarding the serum IL-10 levels, a well-known anti-inflammatory cytokine, the values observed in the responder and non-responder volunteers were similar.

## 3. Discussion

In this brief report, our results demonstrated that the first dose of the CoronaVac vaccine elicited a robust specific IFN-γ response, followed by increased antibody responses, both neutralizing the antibodies and IgG response after the second dose of the same vaccine. Although 90 to 180 days after the vaccination, all these responses dropped, and the booster with the Pfizer vaccine significantly enhanced the cellular and humoral responses in all volunteers. In addition to these results, interestingly, we were able to demonstrate that the two volunteers who showed the worst humoral responses (non-responders) after vaccination with CoronaVac presented a remarkable presence of senescent T cells (CD27−CD28−CD57+), in association with a pro-inflammatory systemic status, seen exclusively in the pre-vaccination sample.

In terms of the initial immune response (both cellular and humoral) found in vaccination with CoronaVac in this case report, our findings were in agreement with the literature, which reported a peak humoral response of this vaccination (IgG response and neutralizing antibodies) 28 days after the administration of the second dose in a population of healthy individuals [12]. Furthermore, it was also demonstrated that the humoral response was reduced 90 days after the second dose. Regarding the cellular response, the authors described that the number of IFN-γ-producing cells to the recombinant S protein from SARS-CoV-2 was higher both 28 and 90 days after the vaccination, even though a remarkable variation in these values was evidenced between the volunteers in these time points, as in this brief report [12]. Similar to the above reported information, Zhao and collaborators (2022) demonstrated that the peak IgG response and neutralizing antibodies were reached 28 days after the second dose of vaccination with CoronaVac and that these levels dropped progressively until 12 weeks. In relation to the cellular response, the numbers of specific IFN-γ-producing T cells were higher 28 days and 3 months after the second dose of CoronaVac, significantly dropping after 6 and 12 months [13]. Adding to these data, it is paramount to highlight that we also observed a rapid and robust response of specific IFN-γ elicited after the first dose of vaccination with CoronaVac.

Concerning the schedule of the vaccination applied in the present study, our findings corroborate the literature, since it was reported that the mean values of IgG response to anti-S1 antigen in a population of healthcare workers significantly increased from the first dose to the second dose, whereas the concentrations significantly decreased after 10 weeks and began to prominently rise after the third booster dose with Pfizer vaccine. Moreover, in the same study, it was demonstrated that both the neutralizing antibody levels and cellular responses, assessed by IFN-γ response, were significantly increased from 10 weeks after the second dose with CoronaVac vaccine to the third booster dose with Pfizer vaccine, in accordance with our results [14].

Despite the findings showing that, in a general way, the CoronaVac vaccine is able to elicit a robust immune response, both after the first and, mainly, after the second dose, and, in the present study, we verified that two volunteers did not respond to the vaccination in the same way as the other volunteers.

In order to understand what factor(s) could be involved in this worse response to vaccination, we initially performed an immunophenotyping evaluation. In fact, it was evidenced that these two volunteers with worse responses presented a higher number of senescent CD8+ or DN T cells, particularly associated with the effector memory profile. Interestingly, the other volunteers presented an elevated number of activated effectors CD4+ T cells, which can not only upregulate CD8+ T cell response, particularly B cells, to produce high affinity, neutralizing antibodies against virus agents [15], but can also directly act as effector cells in antiviral immunity through the production of antiviral cytokines, as well as by direct cytotoxicity [16].

Based on the literature, the occurrence of senescence of T cells can be considered a cornerstone aspect that leads to the impairment of responses to vaccination [17,18]. According to the literature, senescent T cells exhibit downregulation of CD27 and CD28 in association with the upregulation of CD57. Thus, the presence of T cells expressing CD27−CD28−CD57+ indicates that these cells are terminally differentiated senescent-like cells and, interestingly, can compose 30% and 50% of the overall CD8+ T cell compartment in young adults and older adults, respectively [17,19,20]. In addition, it is well-known that CD8+ T cell populations present more characteristics associated with senescent phenotypes, such as the accumulation of differentiated dysfunctional cells, than the CD4+ T population [21]. In terms of COVID-19, it was described that, after primary vaccination for SARS-CoV-2, there is earlier development of CD8+ T cells than antibodies, and these cells may be responsible for the initial vaccine-mediated protection [22]. Furthermore, it is also suggested that the IFN-γ production from CD8+ T cells can enhance both the cellular and humoral immune responses after immunization [23,24], demonstrating that CD8+ T cells are essential for improving not only virus clearance, but also the efficacy of vaccines [21].

Double-negative (DN) T cells (CD3+CD4−CD8−) are a rare and less commonly described subset of peripheral T cells, but play important multifunctional roles in inflammation, cancer, immune disorders, and infection, including virus infection. These cells can recognize viral nucleoproteins and non-protein antigens and produce Th1 and Th2 cytokines and chemokines, as well as presenting cytotoxic activities [25,26]. A recent study showed a high frequency of fully functional viral-specific DN T cells in SARS-CoV-2-infected children [27]. Although the senescent phenotype has been described for CD4 or CD8 T lymphocytes, it is possible that a DN cell with this profile also presents impairments in its proliferative and functional activity, impairing the assembly of an anti-viral response.

Interestingly, until recently, it had been pointed out that T cell-mediated surveillance was maintained through the recirculation of these cells, especially by effector memory T cells [21]. However, it has recently been suggested that the surveillance of T cells occurs mainly by resident memory T cells in different tissues that rarely recirculate between tissues and blood [28]. Thus, the increase in the frequency of effector memory CD8+ T cells with senescent features in the circulation allow us to suggest that these cells were not able to appropriately aid the immune surveillance.

Beyond these facts, in agreement with the literature, double-negative (DN) T cells (CD3+CD4−CD8−), despite being a rare and less described subset of peripheral T cells, play important multifunctional roles in inflammation, cancer, immune disorders, and infection, including virus infection. In this regard, they can recognize viral nucleoproteins and non-protein antigens, produce Th1 and Th2 cytokines, and present cytotoxic activities [25,26]. Of interest, a recent study showed a high frequency of fully functional viral-specific DN T cells in SARS-CoV-2 infected children [27]. As formerly mentioned, although the senescent phenotype has been described for CD4 or CD8 T lymphocytes [21], it is reasonable to suggest that a DN cell with a senescent profile could also present impairments in its proliferative and functional activity, impairing the assembly of a robust anti-viral response.

Following the observation that the two volunteers with worse responses presented a higher number of circulating senescent CD8+ T cells, we evaluated the systemic inflammatory status, since it has been documented that these cells can be influenced by an inflammatory milieu, which can fuel it through the production of many pro-inflammatory molecules, a situation known as a senescence-associated secretory phenotype (SASP) [17,29,30]. Corroborating this information, our results show that the two volunteers presented higher systemic levels of pro-inflammatory cytokines TNF-a and IL-6, with no alteration in the anti-inflammatory cytokine IL-10, when compared to the values observed in the other volunteers. Consequently, this altered the inflammatory status and impacted the pro- and anti-inflammatory ratio, which allows us to also suggest that the imbalance of systemic inflammatory status toward a pro-inflammatory direction could favor the development of senescent T cells.

The presence of an increased systemic pro-inflammatory status found in one volunteer with a worse response to vaccination could be putatively attributed to the high BMI value (>30 kg/m^2^), which indicates obesity, a comorbidity closely associated with a chronic and systemic pro-inflammatory condition [31,32]. In addition, based on the literature, the observation of a raised systemic pro-inflammatory status in the other volunteer, non-obese and aged, could be putatively attributed to the continuous T cell stimulation by antigens from several pathogens, preferentially virus, which can elicit repeated cycles of cell proliferation [30,33].

In an interesting way, it has been evidenced that the presence of an increased number of exhausted and senescent T cells with shorter telomere lengths in COVID-19 patients is related to a severe outcome for COVID-19, in both older and young people [34,35], due to their association with SASP, which can fuel the cytokine storm, since these cells are able to produce large quantities of several pro-inflammatory cytokines, such as IFN-γ and TNF-α [36,37].

The current study presented some limitations, which include: the small number of volunteers enrolled, which, when separated into groups of responders and non-responders, did not allow us to carry out statistical analyses, as well as the number of cytokines assessed. However, despite these limitations, the data presented in this brief report may be useful for increasing the information concerning the immune response elicited after vaccination for COVID-19, particularly in situations where some individuals present a failure to elicit a robust and protective immune response to this vaccination.

## 4. Conclusions

Our results demonstrate that CoronaVac was able to elicit an early cellular response to anti-N and anti-S, followed by an increase in the humoral response, which decreased over time (90 and 180 days after the second dose), and that both cellular and humoral responses increased sharply after the booster dose with messenger RNA vaccine (Pfizer vaccine). In addition, in this report, we also demonstrate that the volunteers with a poor response to COVID-19 vaccination (non-responders) presented both a higher systemic pro-inflammatory status and the presence of senescent T cells, which can putatively impair the immune response to vaccination.

## Figures and Tables

**Table 1:** Response Specific COVID-19 interferon-gamma (IFN-γ) Anti-S and Anti-N (ELISPOT).

Volunteer	Gender	Age	Comorbidity			CoronaVac								Pfizer Booster	
				Pre-Vaccine		Post 1st Dose (30 Days)		Post 2nd Dose (30 Days)		Post 2nd Dose (90 Days)		Post 2nd Dose (180 Days)		Post Dose (25 Days)	
				Anti-S	Anti-N	Anti-S	Anti-N	Anti-S	Anti-N	Anti-S	Anti-N	Anti-S	Anti-N	Anti-S	Anti-N
1	F	49	NONE	0	0	12	≤4	5	≤4	6	≤4	12	6	>20	6
2	M	58	NONE	0	0	≤4	≤4	≤4	≤4	0	≤4	≤4	≤4	>20	≤4
3	F	42	SAH and SLE *	0	0	11	9	≤4	≤4	≤4	≤4	≤4	≤4	13	≤4
4	F	24	NONE	0	0	≤4	7	0	≤4	ND	ND	0	5	9	8
5	F	43	BMI > 30	8	0	>20	>20	>20	>20	>20	7	>20	14	>20	11
6	M	41	ASTHMA	0	0	ND	ND	≤4	6	ND	ND	≤4	6	14	5
7	M	37	NONE	0	0	13	≤4	8	5	≤4	≤4	≤4	≤4	>20	≤4
8	F	46	HYPOTHYROIDISM	0	0	≤4	≤4	≤4	≤4	≤4	≤4	0	6	14	>20

Qualitative detection of specific COVID-19 interferon-gamma (IFN-γ), with S and N panels, showing the pre-vaccine profile and post-vaccination follow-up for each volunteer. SAH: Systemic Arterial Hypertension; SLE: Systemic Lupus Erythematosus (* Volunteer was asymptomatic and without medication); Body Mass Index (BMI). Not Done (ND); Absence of Spots = Zero (0); Non-Reactive Result ≤ 4 spots; Borderline Result: 5, 6 or 7 spots and Reactive Result ≥ 8 spots (Blue).

**Table 2:** Systemic cytokine levels (pg/mL) and the ratio between pro-inflammatory (TNF-α and IL-6) and anti-inflammatory (IL-10) cytokines in the volunteers enrolled in the present study from before the vaccination for COVID-19, both individually and separated into groups of responders and non-responders to the vaccination.

Volunteer	TNF-α (pg/mL)	IL-6 (pg/mL)	IL-10 (pg/mL)	TNF-α/IL-10 Ratio	IL-6/IL-10 Ratio
1	109.23	46.20	11.55	9.46	3.54
2	22.47	7.77	12.35	1.82	0.63
3	5.71	7.73	14.27	0.40	0.54
4	6.98	7.54	11.86	0.59	0.64
5	407.91	43.62	12.34	33.06	4.00
6	11.20	9.17	14.13	0.79	0.65
7	6.47	8.53	12.06	0.54	0.71
8	10.62	9.32	13.73	0.77	0.68
Responder	24.68 ± 8.57	8.31 ± 0.86	13.27 ± 1.09	0.82 ± 0.51	0.64 ± 0.05
Non-responder	258.57 ± 42.12	44.91 ± 1.82	11.95 ± 0.55	21.25 ± 16.68	3.77 ± 0.33

## Data Availability

The authors agree to provide study data upon request.

## References

[1] Gandhi R.T., Lynch J.B., Del Rio C. (2020). Mild or Moderate COVID-19. N. Engl. J. Med..

[2] Pedersen S.F., Ho Y.C. (2020). SARS-CoV-2: A storm is raging. J. Clin. Invest..

[3] World Health Organization (2022). WHO Coronavirus (COVID-19) Dashboard.

[4] Lurie N., Saville M., Hatchett R., Halton J. (2020). Developing COVID-19 Vaccines at Pandemic Speed. N. Engl. J. Med..

[5] Zhang J., Zeng H., Gu J., Li H., Zheng L., Zou Q. (2020). Progress and prospects on vaccine development against SARS-CoV-2. Vaccines.

[6] Lu L., Mok B.W.Y., Chen L.L., Chan J.M.C., Tsang O.T.Y., Lam B.H.S., Chuang V.W.M., Chu A.W.H., Chan W.M., Ip J.D. (2022). Neutralization of Severe Acute Respiratory Syndrome Coronavirus 2 Omicron variant by sera from BNT162b2 or CoronaVac Vaccine Recipients. Clin. Infect. Dis..

[7] Chen Y., Liu Q., Guo D. (2020). Emerging coronaviruses: Genome structure, replication, and pathogenesis. J. Med. Virol..

[8] Ranzani O.T., Hitchings M.D.T., Dorion M., D’Agostini T.L., de Paula R.C., de Paula O.F.P., Villela E.F.D.M., Torres M.S.S., de Oliveira S.B., Schulz W. (2021). Effectiveness of the CoronaVac vaccine in older adults during a gamma variant associated epidemic of COVID-19 in Brazil: Test negative case-control study. BMJ.

[9] Fonseca M.H.G., de Souza T.F.G., Araújo F.M.C., de Andrade L.O.M. (2022). Dynamics of antibody response to CoronaVac vaccine. J. Med. Virol..

[10] Ortega M.M., da Silva L.T., Candido E.D., Zheng Y., Tiyo B.T., Ferreira A.E.F., Corrêa-Silva S., Scagion G.P., Leal F.B., Chalup V.N. (2022). Salivary, serological, and cellular immune response to the CoronaVac vaccine in health care workers with or without previous COVID-19. Sci. Rep..

[11] Escobar A., Reyes-López F.E., Acevedo M.L., Alonso-Palomares L., Valiente-Echeverría F., Soto-Rifo R., Portillo H., Gatica J., Flores I., Nova-Lamperti E. (2022). Evaluation of the immune response induced by CoronaVac 28-day schedule vaccination in a healthy population group. Front. Immunol..

[12] Zhao W., Chen W., Li J., Chen M., Li Q., Lv M., Zhou S., Bai S., Wang Y., Zhang L. (2022). Status of humoral and cellular immune responses within 12 months following CoronaVac vaccination against COVID-19. Vaccines.

[13] Hayashi J.Y., Simizo A., Miyamoto J.G., Costa L.V.S., Souza O.F., Chiarelli T., Bacarov N.B., Hidalgo R., Garcia L.D., Soane M.M. (2022). Humoral and cellular responses to vaccination with homologous CoronaVac or ChAdOx1 and heterologous third dose with BNT162b2. J. Infect..

[14] Sant A.J., McMichael A. (2012). Revealing the role of CD4^+^ T cells in viral immunity. J. Exp. Med..

[15] Swain S.L., McKinstry K.K., Strutt T.M. (2012). Expanding roles for CD4^+^ T cells in immunity to viruses. Nat. Rev. Immunol..

[16] Hirai T., Yoshioka Y. (2022). Considerations of CD8+ T cells for optimized vaccine strategies against respiratory viruses. Front. Immunol..

[17] Tedeschi V., Paldino G., Kunkl M., Paroli M., Sorrentino R., Tuosto L., Fiorillo M.T. (2022). CD8^+^ T cells senescence: Lights and shadows in viral infection, autoimmune disorders and cancer. Int. J. Mol. Sci..

[18] Agrawal A., Weinberger B. (2022). Editorial: The impact of immunosenescence and senescence of immune cells on responses to infection and vaccination. Front. Aging.

[19] Lian J., Yue Y., Yu W., Zhan Y. (2020). Immunosenescence: A key player in cancer development. J. Hematol. Oncol..

[20] Pangrazzi L., Reidla J., Arana J.A.C., Naismith E., Miggitsch C., Meryk A., Keller M., Krause A.A.N., Melzer F.L., Trieb K. (2020). CD28 and CD57 define four populations with distinct phenotypic properties within human CD8^+^ t cells. Eur. J. Immunol.

[21] Oberhardt V., Luxenburger H., Kemming J., Schulien I., Ciminski K., Giese S., Csernalabics B., Lang-Meli J., Janowska I., Staniek J. (2021). Rapid and stable mobilization of CD8^+^ T cells by SARS-CoV2 mRNA vaccine. Nature.

[22] Li C., Lee A., Grigoryan L., Arunachalam P.S., Scott M.K.D., Trisal M., Wimmers F., Sanyal M., Weidenbacher P., Feng Y. (2022). Mechanisms of innate and adaptative immunity to the Pfizer-BioNTech BNT162b2 vaccine. Nat. Immunol..

[23] Borriello F., Poli V., Shrock E., Spreafico R., Liu X., Pishesha N., Carpenet C., Chou J., Di Gioia M., McGrath M.E. (2022). An adjuvant strategy enabled by modulation of the physical properties of microbial ligands expands antigen immunogenicity. Cell.

[24] Wu Z., Zheng Y., Sheng J., Han Y., Yang Y., Pan H., Yao J. (2022). CD3+CD4-CD8- (Double-Negative) T cells in inflammation, immune disorders and cancer. Front. Immunol..

[25] Sundaravaradan V., Saleem R., Micci L., Gasper M.A., Ortiz A.M., Else J., Silvestri G., Paiardini M., Aitchison J., Sodora D. (2013). Multifuncional double-negative T cells in sooty mangabeys mediate T-helper functions irrespective of SIV infection. PLoS Pathog..

[26] Hsieh Li-En Song J., Grifoni A., Shimizu C., Tremoulet A.H., Dummer K.B., Burns J.C., Sette A., Franco A. (2022). T cells in multisystem inflammatory syndrome in children (MIS-C) have a predominant CD4+ T Helper response to SARS-CoV-2 peptides and numerous virus-specific CD4-CD8- Double-Negative T cells. Int. J. Mol. Sci..

[27] Steinert E.M., Schenkel J.M., Fraser K.A., Beura L.K., Manlove L.S., Igyártó B.Z., Southern P.J., Masopust D. (2015). Quantifying memory CD8 T cells reveals regionalization of immunosurveillance. Cell.

[28] Callender L.A., Carroll E.C., Beal R.W.J., Chambers E.S., Nourshargh S., Akbar A.N., Henson S.M. (2017). Human CD8+ EMRA T cells display a senescence-associated secretory phenotype regulated by p38 MAPK. Aging Cell.

[29] Lopes-Paciencia S., Saint-Germain E., Rowell M.-C., Ruiz A.F., Kalegari P., Ferbeyre G. (2019). The senescence-associated secretory phenotype and its regulation. Cytokine.

[30] Caci G., Albini A., Malerba M., Noonan D.M., Pochetti P., Polosa R. (2020). COVID-19 and Obesity: Dangerous Liaisons. J. Clin. Med..

[31] Aghili S.M.M., Ebrahimpur M., Arjmand B., Shadman Z., Sani M.P., Qorbani M., Larijani B., Payab M. (2021). Obesity in COVID-19 era, implications for mechanisms, comorbidities, and prognosis: A review and meta-analysis. Int. J. Obes..

[32] Strioga M., Pasukoniene V., Characiejus D. (2011). CD8+ CD28- and CD8+CD57+ T cells and their role in health and disease. Immunology.

[33] Sanchez-Vazquez R., Guío-Carríon A., Zapatero-Gaviria A., Martínez P., Blasco M.A. (2021). Shorter telomere lengths in patients with severe COVID-19 disease. Aging.

[34] Wang Q., Codd V., Raisi-Estabragh Z., Musicha C., Bountziouka V., Kaptoge S., Allara E., Angelantonio E.D., Butterworth A.S., Wood A.M. (2021). Shorter leokocyte telomere length is associated with adverse COVID-19 outcomes: A cohort study in UK Biobank. eBioMedicine.

[35] Zheng H.Y., Zhang M., Yang C.X., Zhang N., Wang X.C., Yang X.P., Dong X.Q., Zheng Y.T. (2020). Elevated exhaustion levels and reduced functional diversity of T cells in peripheral blood may predict severe progression in COVID-19 patients. Cell. Mol. Immunol..

[36] De Biasi S., Meschiari M., Gibellini L., Bellinazzi C., Borella R., Fidanza L., Gozzi L., Iannone A., Tartaro D., Mattioli M. (2020). Market T cell activation, senescence, exhaustion and skewing towards TH17 in patients with COVID-19 pneumonia. Nat. Commun..

[37] Diao B., Wang C., Tan Y., Chen X., Liu Y., Ning L., Chen L., Li M., Liu Y., Wang G. (2020). Reduction and functional exhaustion of T cells in patients with Coronavirus Disease 2019 (COVID-19). Front. Immunol..

